# Molecular Mechanisms Regulating Obesity-Associated Hepatocellular Carcinoma

**DOI:** 10.3390/cancers12051290

**Published:** 2020-05-20

**Authors:** Yetirajam Rajesh, Devanand Sarkar

**Affiliations:** Department of Human and Molecular Genetics, Massey Cancer Center, VCU Institute of Molecular Medicine (VIMM), Virginia Commonwealth University, Richmond, VA 23298, USA; Rajesh.Yetirajam@vcuhealth.org

**Keywords:** HCC, obesity, NASH, genetic factors, epigenetic changes, therapeutics

## Abstract

Obesity is a global, intractable issue, altering inflammatory and stress response pathways, and promoting tissue adiposity and tumorigenesis. Visceral fat accumulation is correlated with primary tumor recurrence, poor prognosis and chemotherapeutic resistance. Accumulating evidence highlights a close association between obesity and an increased incidence of hepatocellular carcinoma (HCC). Obesity drives HCC, and obesity-associated tumorigenesis develops via nonalcoholic fatty liver (NAFL), progressing to nonalcoholic steatohepatitis (NASH) and ultimately to HCC. The better molecular elucidation and proteogenomic characterization of obesity-associated HCC might eventually open up potential therapeutic avenues. The mechanisms relating obesity and HCC are correlated with adipose tissue remodeling, alteration in the gut microbiome, genetic factors, ER stress, oxidative stress and epigenetic changes. During obesity-related hepatocarcinogenesis, adipokine secretion is dysregulated and the nuclear factor erythroid 2 related factor 1 (Nrf-1), nuclear factor kappa B (NF-κB), mammalian target of rapamycin (mTOR), phosphatidylinositol-3-kinase (PI3K)/phosphatase and tensin homolog (PTEN)/Akt, and Janus kinase/signal transducer and activator of transcription (JAK/STAT) signaling pathways are activated. This review captures the present trends allied with the molecular mechanisms involved in obesity-associated hepatic tumorigenesis, showcasing next generation molecular therapeutic strategies and their mechanisms for the successful treatment of HCC.

## 1. Introduction

Obesity is of momentous concern worldwide with substantial risk factors for several types of cancer. It is characterized by the accumulation of excess body fat that is harmful to health, defined by the body mass index (BMI) by the World Health Organization (WHO) [1]. Normal BMI values lie in the range of 18.5–24.9 kg/m^2^; for overweight, it is 25–29.9 kg/m^2^, and lean refers to weights below 18.5 kg/m^2^. Accordingly, in the USA, around two-thirds of the population aged >20 years are currently overweight, with an approximately 35% prevalence of obesity [2]. This might increase up to 42% by 2030, especially in people >18 years [3]. The main driving forces towards increasing obesity are an overall increase in caloric intake with sedentary snacking patterns involving high carbohydrate beverages and dietary fat [4], low physical activity and the significant but incompletely deciphered role of genetic factors [5]. Substantial consequences of obesity include the medical and socio-economic burdens of obesity-related comorbidities, such as coronary heart disease, type-2 diabetes mellitus, respiratory disease and cancer [6]. Several studies also reveal direct correlations between environmental factors and diet, nutrition, obesity and cancer [1]. Epidemiological studies display a direct association between obesity and hepatocellular carcinoma (HCC) [7]. However, the mechanisms relating obesity and HCC are still being unraveled and may include adipose tissue remodeling, insulin resistance (IR) and other metabolic disorders [8]. Henceforth, this review majorly focuses on the molecular signaling pathways involved in obesity-associated HCC.

## 2. Hepatocellular Carcinoma and Obesity

HCC, arising from the hepatocytes, is the most common liver cancer. It is also the sixth most common cancer in terms of incidence [9]. HCC has been recognized as the second most common cause of cancer-related deaths in males globally [9]. Studies also reflect lack of therapeutic options for the majority of HCC cases. The single most significant risk factor associated with HCC is liver cirrhosis [10]. There are a number of underlying etiologies, all causing chronic inflammation, leading towards HCC, the most common being viral hepatitis caused by the hepatitis B virus (HBV) and hepatitis C virus (HCV). Additional causes include alcoholism, aflatoxins and metabolic disorders like hemochromatosis. It has been noticed that globally, HCC-related mortality is rising prominently, and surprisingly, the increase is irrespective of the prevalence of viral hepatitis. Evidence over a decade highlights a close correlation between obesity and liver cancer in northern European [11] and American populations [12]. It has been also comprehended that obesity and impaired glucose tolerance are the most contributory factors in two-thirds of HCC individuals [13,14].

A study by Calle et al. documented a landmark epidemiology linking 14% of cancer-related deaths in women and 20% in men in USA to being overweight or obese [12]. In this study, according to BMI, the relative risks (RR) of cancer of >900,000 individuals were stratified. The RR of liver cancer deaths increased with BMIs, reaching up to 4.52 in those with a BMI >35, in comparison with normal weight individuals. In some cases, the RR was undoubtedly raised lacking clear documentation for the underlying reason, and the probable reasons were considered to be liver malfunctioning/obesity/impaired glucose tolerance. Consequential studies based in Asia and Europe also deciphered a fundamental role of obesity in HCC risk, either individually or as a cofactor [15].

## 3. Non-Alcoholic Steatohepatitis (NASH): A Precursor of Obesity-Associated HCC

Obesity-associated HCC is always preceded by non-alcoholic fatty liver disease (NAFLD), a spectrum extending from non-alcoholic fatty liver (NAFL)—defined by lipid accumulation in the hepatocytes exceeding 5% of the liver weight in the absence of viral hepatitis, alcoholism and hereditary disorders—to the advanced condition non-alcoholic steatohepatitis (NASH), the latter encompassing hepatic steatosis (fatty liver), inflammation, hepatocyte injury and a varying degree of fibrosis [14]. However, these patients display substantial heterogeneity, and metabolic (dysfunction) associated fatty liver disease (MAFLD) has been suggested to be a more appropriate overarching term to describe these patients [16]. A meta-analysis of 86 studies on 8.5 million subjects from 22 countries reveal that the global prevalence of NAFLD is 25.24% [17]. In the USA, in 2015, 83.1 million people (~25% of the population) was diagnosed with NAFLD, and it is expected to increase to 100.9 million in 2030, with 20–27% of adults with NAFLD developing NASH [18]. In most cases, especially in North America and the USA, NAFLD is associated with obesity, although in Asia, a condition called “lean NASH” is observed in which NAFLD develops in people with normal BMIs, indicating a role of genetic factors in developing NASH and hence associated HCC [19]. Among all the HCC cases, in separate studies, a range of 4–22% of cases have been shown to be associated with NASH [17].

The predominant contributing factors for obesity are overnutrition and physical inactivity, which are compounded by genetic variations. NAFLD and NASH develop when the capacity of the liver to handle carbohydrates and lipids is overwhelmed because of obesity [14]. Circulating free fatty acid (FFA) levels are increased in obesity, becoming available to the liver. A second source of FA in the liver is *de novo* lipogenesis (DNL), employing excess dietary carbohydrate, especially fructose, as a substrate [20]. The enzymes regulating DNL, such as acetyl-CoA carboxylase (ACC), are under the transcriptional control of sterol regulatory element-binding protein 1c (SREBP-1c) and carbohydrate regulatory-binding protein (ChREBP), also known as MLXIPL. In the liver, FAs can either be re-esterified into triglycerides (TG) and stored as lipid droplets or undergo β-oxidation in mitochondria and peroxisomes to generate energy. In obesity, some of the excess FAs are converted to TG and stored as lipid droplets, while the rest burden the mitochondrial capacity for oxidizing FA, with the generation of reactive oxygen species (ROS) and toxic lipids, like ceramides, that damage the liver and induce an inflammatory response, leading to NASH. ROS and toxic lipids cause hepatocyte injury by engaging a variety of mechanisms, such as endoplasmic reticulum (ER) stress with an unfolded protein response (UPR), the induction of apoptosis, and an augmented wound healing response because of the activation of nuclear factor kappa B (NF-κB) and inflammasomes, causing inflammation, and these processes are aggravated by external factors, such as cytokines and adipokines, hypoxia and, very importantly, products of the gut microbiome [14,21]. When this chronic inflammatory process with cell death, compensatory proliferation and wound healing continues unabated for decades, it creates a milieu where DNA damage-induced mutations ultimately cause HCC [21].

DNA damage plays an important role in HCC, and n-nitrosodiethylamine (DEN) is a DNA damaging hepatocarcinogen, which is frequently used to establish mouse models of HCC. ROS and reactive nitrogen species (RNS) are generated by chronic inflammation in NASH, and NASH patients show higher levels of oxidative DNA damage, and these levels were further augmented in NASH-HCC patients [22]. While ROS are generated as a by-product of metabolism, especially β-oxidation, by the hepatocytes, ROS produced by the recruited macrophages and neutrophils create additional damage, leading to carcinogenesis [23]. It is the combination of oxidative damage with compensatory proliferation stimulated by oncogenes that ultimately leads to HCC development. Transgenic mice with the hepatocyte-specific expression of the oncogene URI (unconventional prefoldin RBP5 interaction) developed DNA damage because of the inhibition of enzymes regulating NAD metabolism leading to HCC, and when fed a high fat diet (HFD), these mice developed NASH and, subsequently, HCC, which was associated with T helper 17 (Th17) lymphocyte-mediated inflammation [24,25]. The dysregulation of DNA damage response (DDR) genes thus might play a role in NASH and HCC. DNA-PK, which mediates DNA damage repair by nonhomologous end joining, was shown to promote fatty acid synthase expression, and its expression was found to be higher in HCC [26,27]. However, in-depth in vivo studies are lacking to convincingly establish the role of DDR genes in NASH and HCC.

Here, we will provide a comprehensive review of the genetic and epigenetic factors and pathogenic pathways and processes that predispose to the development of NAFLD and/or progression to HCC.

## 4. Insights into Molecular Mechanisms Promoting Obesity-Associated HCC

### 4.1. Genetic Factors

Genome wide association studies (GWAS) have recognized >175 obesity associated genomic loci [28]. Advancements in the genetic technology highlighting the delineation of single nucleotide changes have revealed the molecular mechanisms of weight regulation. Few known genetic aberrations have been identified by the high throughput sequencing of exomes/genomes or target sequencing in individuals/cohorts of adults/children. These studies provide insight into the pathophysiology of weight regulation, identify genetic and epigenetic modifications playing a significant role in weight gain and also unravel potential treatments in selected individuals [29]. The genetic causes of obesity could be broadly classified into monogenic, syndromic and polygenic. Monogenic causes majorly comprise of single gene mutations predominantly located in the leptin-melanocortin pathway. Generally, mutations require two alleles of gene dysfunction in homozygous/heterozygous form for phenotypic changes. Syndromic obesity refers to phenotypic obesity such as neurodevelopmental and other organ/system malfunctioning and the early onset of severe obesity. This results from changes in a single gene or larger chromosomal region including numerous genes. Polygenic obesity involves a cumulative number of genes facilitating a weight gain promoting environment [29]. Thus, there are many genetic factors contributing to obesity. However, not all lead to the development of NASH and HCC. Here, we will highlight those genetic factors that have been identified to play a role in NASH and HCC either by GWAS in patients, by the analysis of mouse models, or both.

#### 4.1.1. Patatin Like Phospholipase Domain Containing 3 (PNPLA3)

GWAS identified the association of the rs738409 C > G single nucleotide polymorphism (SNP) in the PNPLA3 gene with NAFLD, which has been validated by many subsequent studies [30,31]. This polymorphism results in the Ile148Met variant protein, and individuals carrying this mutation have a three-times higher risk for developing NASH and a 12-times increased risk for developing HCC when compared to non-carriers [32]. PNPLA3 regulates the lipolysis of lipid droplets in hepatocytes, and the Ile148Met variant is resistant to proteasomal degradation, accumulating on lipid droplets and preventing lipolysis, and a knock-in mouse expressing physiological levels of this variant developed steatosis when fed a high sucrose diet [33,34,35]. However, the knock-in mouse was not followed for a long period of time to determine whether it developed HCC. Primary human hepatic stellate cells (HSC) harboring this PNPLA3 variant showed augmented fibrogenic activation associated with c-Jun NH2 terminal kinase (JNK)-mediated phosphorylation and the inactivation of peroxisome proliferator activated receptor γ (PPARγ) [36]. Knocking out PNPLA3 showed no effect on metabolism in mice, further confirming that the mutation confers a new function to the protein contributing to steatosis [37].

#### 4.1.2. Transmembrane 6 Superfamily Member 2 (TM6SF2)

An exome-wide association study identified a significant association of polymorphism rs58542926 c.449 C > T, p.Glu167Lys in the TM6SF2 gene with liver fat content and circulating levels of the liver injury marker alanine transaminase (ALT), with unexpectedly lower levels of serum TG, low-density lipoprotein cholesterol and total cholesterol [38]. A study on 3556 participants from the Amish Complex Disease Research Program carrying the TM6SF2 polymorphism and employing zebrafish models with TM6SF2 deficiency unraveled a potential role of TM6SF2 in regulating lipid absorption and metabolism in the small intestine, resulting in decreased postprandial serum TG [39]. In mice, the knockdown of TM6SF2 by adeno-associated virus (AAV)-delivered shRNA increased hepatic TG levels and, upon feeding high sucrose diet, led to the development of steatosis [38]. Using a primary human hepatocytes 3D spheroid model, it was documented that the TM6SF2 E167K variant increases intracellular fat content by decreasing the secretion of Apolipoprotein B (APOB) particles [40]. TM6SF2 was demonstrated to localize in the ER, and knocking down TM6SF2 in human HCC cells resulted in a decrease in the levels of TG synthesis genes, while increasing the lipid droplet content and TM6SF2 overexpression increased lipid droplets [41]. TM6SF2 knockdown did not affect the viability and proliferation of human HCC cells [41]. A meta-analysis encompassing 6873 patients identified a significant association of TM6SF2 polymorphism with HCC risk [42]. As yet, the function of TM6SF2 is not known, and the molecular mechanism by which it regulates steatosis and HCC remains to be determined.

#### 4.1.3. Hydroxysteroid 17-Beta Dehydrogenase 13 (HSD17B13)

A GWAS study using a total of 37,173 patients identified a highly significant association between a splice variant (rs72613567:TA) in HSD17B13 with reduced levels of ALT and AST, and a reduced risk for NASH [43]. HSD17B13 encodes a hepatic lipid droplet protein that uses nicotinamide adenosine dinucleotide as a cofactor for its enzymatic activity, and the presence of this variant resulted in the formation of a truncated protein with reduced enzymatic activity. It was hypothesized that in patients with fatty liver, this truncated protein protects from hepatocyte injury, especially in the presence of the PNPLA3 pI148M variant [43]. A comparative study between carriers and non-carriers of the rs72613567:TA variant identified decreased fibrosis and inflammatory gene levels in the carriers [44]. This variant predicted a reduced risk of developing HCC [45]. HSD17B13 was identified to be downregulated in HCC patients, and the overexpression of HSD17B13 induced G_1_ arrest in human HCC cells [46]. A NASH genetic risk score (GRS), combining the variants in the PNPLA3, TM6SF2 and HSD17B13 genes, predicted a 12-fold increased risk for cirrhosis and a ~29-fold increased risk for HCC [47]. Clinical trials using HSD17B13 RNAi have been initiated in NASH patients (https://www.clinicaltrialsarena.com/news/arrowhead-doses-first-patient-aro-hsd/). HSD17B13 is still a relatively under-studied gene, with little understanding of its molecular mechanism of action, and more in-depth studies are required to decipher the consequence of HSD17B13 inhibition.

### 4.2. Epigenetic Factors

Epigenetic studies mainly consider the heritable regulatory changes in gene expression devoid of alterations in the nucleotide sequence [48]. These modifications consider the differential packaging of DNA that either promotes or inhibits the expression of certain genes. The epigenetic programming of parental gametes, the fetus and postnatal development are commonly under the influence of environmental and dietary factors or the gut microbiome [49]. Studies have identified many changes in the methylation of DNA and histones, the acetylation of histones, and non-coding RNA (ncRNA)-mediated gene regulation in patients with NAFLD or HCC or in mouse models of NAFLD. We will focus, here, only on those modifications that have shown to contribute to the spectrum of NASH to HCC in an in vivo model.

#### 4.2.1. Dysregulation of Methylation

The addition of methyl groups to the cytosine at CpG sites, more commonly present in the promoter regions in the genes, is a major mechanism of gene regulation, and hypermethylation is usually associated with gene silencing. DNA methylation profile analysis reveals that functionally relevant differences in methylation can differentiate mild NAFLD patients from advanced patients [50]. S-adenosinelmethione (SAMe) is a major methyl donor that is synthesized from methionine by methionine adenosyltransferase (MAT) and catabolized to S-adenosyl-L-homocysteine by glycine N-methyltransferase (GNMT) [51]. A methionine and choline deficient diet is an established mouse model of NASH, and methyl donor supplementation could prevent the progression of HFD-induced NAFLD in mice and performed as a chemopreventive strategy for HCC development in rats [52,53]. GNMT expression is significantly downregulated in the livers of cirrhotic and HCC patients, and GNMT knockout mouse developed NASH and HCC, which was associated with the activation of the Ras and Janus kinase/signal transducer and activator of transcription (JAK/STAT) signaling pathways because of the hypermethylation of the promoters of Ras association domain family member 1 (RASSF1) and suppressor of cytokine signaling 2 (SOCS2), which inhibit these pathways, respectively [54]. Further studies identified a role of the Ras-mediated activation of Serine/threonine kinase 11 (STK11/LKB1) in this process [55]. MAT1A expression is downregulated in cirrhotic and HCC patients, and Mat1a knockout mice developed NASH and HCC associated with the dysregulation of very low-density lipoprotein assembly and ER stress [56,57,58]. It is anticipated that Mat1a knockout would reduce the levels of SAMe and alter the methylation patterns of genes. As yet, gene targets mediating these effects have not been identified, although a potential role of the chaperone protein prohibitin 1 (PHB1) has been suggested [59]. Knocking out betaine-homocysteine S-methyltransferase (BHMT), which catalyzes the conversion of homocysteine to methionine, was associated with a decrease in hepatic SAMe and corresponding increase in S-adenosyl-L-homocysteine, and led to the development of NASH and HCC [60]. Thus, the perturbation of one carbon metabolism, regulated by the methionine cycle, plays a role in NASH pathogenesis and eventual progression to HCC.

In a streptozotocin/HFD-induced (STAM) NASH-HCC mouse model, DNA methylation profiles were analyzed in control, steatotic (6 weeks), NASH-fibrotic (12 weeks) and HCC (20 weeks) livers and correlated with gene expression status [61]. Although progressive changes in DNA methylation or in transcript expression levels were observed for many genes, an inverse correlation between gene expression and gene-specific methylation was observed only for tubulin beta 2B class IIb (Tubb2b), a microtubule cytoskeleton gene. TUBB2B overexpression was identified in human HCC samples, and the oleic acid treatment of HepaRG cells induced TUBB2B expression with a corresponding decrease in TUBB2B CpG methylation [61]. The mechanism by which TUBB2B promotes NASH–HCC remains to be determined.

Unlike DNA methylation, histone methylation has a variable effect on transcription, e.g., the trimethylation of histone H3 at lysine 4 (H3K4me3) indicates active transcription, while demethylation of histone H3 lysine 9 (H3K9me2) is a marker for transcriptional silencing [62]. Lysine-specific demethylase-1 (LSD1/KDM1A) induces the demethylation of H3K9 to activate target gene expression, while the demethylation of H3K4 suppresses transcription [62]. Snail family transcriptional repressor 2 (SNAI2), also known as Slug, is a master regulator of the transcription of many genes conferring epithelial–mesenchymal transition (EMT) and metastasis and is overexpressed in many cancers, including HCC [63]. It was demonstrated that Slug is upregulated by insulin treatment or by feeding in mice [64]. Slug recruits Lsd1 to the fatty acid synthase (Fasn) promoter to induce H3K9 demethylation and increase Fasn transcription and *de novo* lipogenesis. Slug overexpression induced steatosis in mice, and the hepatocyte-specific deletion of Slug protected from HFD-induced steatosis [64]. Although these mice were not followed up regarding the development of NASH–HCC, considering the pivotal role Slug plays in tumorigenesis, it is likely that Slug might play a role in the transition from NASH to NASH-HCC.

#### 4.2.2. Dysregulation of Acetylation

While DNA methylation silences transcription, histone acetylation promotes chromatin decondensation and transcription, which is regulated by histone acetyltransferases (HATs) and histone deacetylases (HDACs). There are four classes of HDACs: zinc-dependent HDACs are class I (HDAC1–3 and HDAC8), class II (HDAC4–10) and class IV (HDAC11), and sirtuins, which are NAD+-dependent protein deacetylases, belong to class III (SIRT1–7) [65]. The expression profiling of 115 chromatin regulators in tumor and adjacent non-tumor tissues from HFD-induced and leptin receptor-deficient (db/db) obese mice identified the overexpression of HDAC8 in tumor tissues, which was confirmed in NAFLD-associated HCC patient samples [66]. HDAC8 was directly upregulated by SREBP-1, and lentivirus-mediated HDAC8 silencing reversed insulin resistance and abrogated NAFLD-associated HCC in mice. HDAC8 overexpression inhibited p53/p21-facilitated apoptosis and G_2_/M cell-cycle arrest and promoted β-catenin-mediated cell proliferation. HDAC8 interacted with chromatin modifier enhancer of zeste 2 polycomb repressive complex 2 subunit (EZH2) to repress wingless/integrated (Wnt) antagonists through histone H4 deacetylation and H3 lysine 27 trimethylation [66]. The hepatocyte-specific deletion of HDAC3 in mice resulted in steatosis, which was associated with the rerouting of metabolic precursors to TG synthesis and storage in lipid droplets and an increase in lipid droplet coat protein perilipin 2, which mediated these effects [67]. Hepatocytes isolated from these mice maintained insulin sensitivity, and overall body weight was not changed. This is not unlikely, because in obesity-induced steatosis, it is insulin resistance in adipocytes that contributes to steatosis. It was documented that HDAC3 is downregulated in mice, and further analysis of hepatocyte-specific HDAC3 knockout mouse revealed the development of spontaneous HCC resulting from increased DNA damage because of the hyperacetylation and consequent demethylation of H3K9, resulting in the increased transcription of genes regulating oncogenic signaling pathways [68].

Mice with a hepatocyte-specific deletion of SIRT1 developed NASH upon feeding a HFD. SIRT1 directly interacts with PPARα and increases PPARα-dependent gene expression. The deletion of SIRT1 blocks PPARα signaling and, therefore, FA β-oxidation [69]. On the contrary, SIRT1 transgenic mice were protected from HFD-induced steatosis with the induction of antioxidant proteins—superoxide dismutase 2 (SOD2/MnSOD) and nuclear factor, erythroid 2 like 1 (NFE2L1/NRF1)—and inhibition of NF-κB activity, resulting in the decreased production of pro-inflammatory cytokines, and they were also protected from DEN-induced DNA damage and DEN/HFD-induced hepatocarcinogenesis [70,71]. SIRT1 levels were decreased in the livers of NAFLD patients, SIRT1 knockdown in human induced pluripotent stem cells (iPSCs)-derived hepatocytes increased fatty acid biosynthesis, and the implantation of these cells in decellularized rat livers resulted in steatosis and inflammation similar to that in human fatty livers [72]. However, SIRT1 has been identified to be overexpressed in HCC and function as an oncogene, indicating that its role in HCC may be context- and etiology-specific [73]. Additionally, although shown effective in mice, human clinical trials were inclusive in deciphering the benefits of SIRT1 activator resveratrol in treating NAFLD [74]. Interestingly, it was documented that SIRT1, in a complex with Forkhead box O3a (FOXO3a) and NRF1, binds to the SIRT6 promoter and promotes its transcription. The hepatocyte-specific deletion of SIRT6 in mice caused increased glycolysis and TG synthesis and decreased FA β-oxidation with the induction of NAFLD by modulating the expression of a variety of genes via H3K9 hyperacetylation, as well as by inducing oxidative stress by downregulating NRF2 [75,76]. The livers of NASH patients showed decreased levels of SIRT6. SIRT6 is downregulated in cirrhosis, and HCC and Sirt6-/- hepatocytes expressed an HCC gene signature [77]. A novel SIRT6 activator, MDL-800, has been identified, which showed efficacy in a xenograft model of human HCC, paving the way for the further evaluation of this agent to treat NASH and NASH-HCC [78].

#### 4.2.3. Non-Coding RNAs (ncRNAs)

ncRNAs are an abundant group of RNA transcripts that do not translate into proteins but make potentially functional RNAs [79]. ncRNAs longer than 200 nucleotides are designated as long ncRNAs (lncRNAs), whereas those that are shorter are termed small ncRNAs (sncRNAs), which include microRNAs (miRNAs), piwi-associated RNAs and small nucleolar RNAs (snoRNAs). ncRNAs are important regulators of epigenetic gene regulation by inducing heterochromatin formation, histone modification, DNA methylation targeting and gene silencing. In a STAM NASH-HCC mouse model, miRNA expression profiles were analyzed in control, steatotic, NASH-fibrotic and HCC livers, demonstrating progressive alterations of hepatic miRNAs with disease development [80]. At steatotic, NASH-fibrotic and HCC stages, 19, 22 and 29 miRNAs were found to be differentially expressed, respectively, among which miR-221-3p, miR-222-3p and miR-223-3p showed progressing stage-dependent increases accompanied by the activation of many oncogenic signaling pathways. Here, we highlight a number of ncRNAs for which the functional roles in NASH and HCC are well-established.

##### miR-122

miR-122 is a highly abundant liver-specific miRNA accounting for 70% of the total miRNAs in the liver and is downregulated in ~70% of human HCC [81]. Knocking out miR-122 in mice resulted in steatohepatitis and HCC with profound alterations of a plethora of genes regulating lipid metabolism, inflammation and fibrosis, and the AAV-mediated delivery of miR-122 markedly inhibited Myc-driven HCC in mice, thereby establishing both the tumor suppressor function of miR-122 and its therapeutic utility [82,83]. Analysis of the liver transcriptome after the deletion of miR-122 at multiple timepoints revealed a widespread deregulation of hepatic transcription, including progressive increases in the expression of imprinted genes, such as those in Igf2 and Dlk1-Dio3 clusters, providing insights into the mechanism by which miR-122 functions as a tumor suppressor [84]. Argonaute-CLIP sequencing in humans and mice identified novel miR-122 targets, such as B cell lymphoma 9 (BCL9), solute carrier family 25 member 2 (SLC52A2) and syntaxin 6 (STX6), which could predict survival in HCC patients [85]. A liver-targeted oncolytic herpes simplex virus (HSV) delivering miR-122 showed strong in vivo efficacy in HCC xenograft models [86]. However, in adult normal mice, an antisense oligonucleotide (ASO)-mediated inhibition of miR-122 decreased plasma cholesterol levels, increased hepatic fatty acid oxidation, and decreased hepatic fatty acid and cholesterol synthesis rates, and in a diet-induced obesity model, the ASO decreased plasma cholesterol levels and improved NAFLD, with the downregulation of lipogenic genes, such as FASN, SREBP1 and stearoyl-coenzyme A desaturase 1 (SCD1). These discrepant observations in phenotypes between the acute versus chronic downregulation of miR-122 require further analysis of its function, especially in regulating lipid metabolism.

##### miR-21

miR-21 is overexpressed in many cancers, functioning as an oncogene [79]. miRNA microarray analysis identified miR-21 to be the most highly overexpressed miRNA in human HCC, and it was demonstrated that it augments the proliferation and invasion of human HCC cells by targeting phosphatase and tensin homolog (PTEN), a negative regulator of the oncogenic phosphatidylinositol-3-kinase/AKT serine/threonine kinase (PI3K/AKT) pathway [87]. miR-21 expression was increased in the livers of high fat diet (HFD)-fed mice, and the knockdown of miR-21 abrogated lipid accumulation in these mice [88]. The transcriptional repressor HMG-box transcription factor 1 (HBP1) was identified as a miR-21 target resulting in an increased expression of p53, leading to cell cycle arrest, and the decreased expression of the p53 target gene SREBP1C, leading to decreased lipogenesis [88]. It was suggested that the inhibition of miR-21 could be a potential treatment strategy both for HCC and for its precursor condition NAFLD.

##### miR-221/222

A comparison between HCC tissues with normal liver and precancerous cirrhotic liver identified miR-221/222 as one of the 12 miRNAs showing significant diagnostic value, and the overexpression of miR-221 increased tumorigenicity in p53-/-, myc-expressing liver progenitor cells [89]. miR-221/222 showed a progressive increase during NASH-HCC development, and the hepatocyte-specific deletion of miR-221/222 ameliorated NASH induced by a methionine and choline deficient diet (MCDD) and CCl_4_ treatment, while the overexpression of miR-221/222 aggravated the phenotype [80,90]. Treatment with antimiRs of miR-221/222 protected from MCDD-induced NASH and significantly reduced the orthotopic xenograft growth of human HCC cells [90,91]. miR-221/222 targets cell cycle regulators, such as p27 and p57; pro-apoptotic proteins, such as BCL2 modifying factor (BMF); modulators of mammalian target of rapamycin (mTOR) pathway, such as DNA damage-inducible transcript 4 (DDIT4); and epigenetic regulators, such as HDAC6, all of which might contribute to its ability to induce NASH and HCC [89,92,93,94].

##### lncRNAs

With the advancement of RNA sequencing technology, many lncRNAs are being identified, and their roles in NASH and HCC are being elucidated [79,95,96]. Recent studies unravel potential roles of lncRNAs in NASH-HCC, which require further validation [97,98,99,100]. lncRNA regulator of Akt signaling associated with HCC and RCC (lncARSR) was upregulated in NAFLD livers in mice and in FA-treated HepG2 cells [97]. lncARSR interacts with Yes1 associated transcriptional regulator (YAP1) and blocks its phosphorylation, which contributes to the activation of the insulin receptor substrate 2 (IRS2)/AKT pathway. lncARSR overexpression increased proliferation, invasion and lipid accumulation in FA-treated HepG2 cells, and lncARSR knockdown abrogated the growth of HepG2 xenografts in HFD-fed nude mice [97]. lncRNA small nucleolar RNA host gene 20 (SNHG20) silencing suppressed M1 polarization in macrophages and delayed the progression of NASH to HCC in a DEN/HFD-fed mouse model [98]. lncRNA nuclear paraspeckle assembly transcript 1 (NEAT1) sponges miR-124-3p, resulting in the increased expression of its target adipose triglyceride lipase (ATGL) [99]. ATGL increased FFA levels, both ATGL and NEAT1 were shown to be overexpressed in HCC patients, and their roles in promoting hepatocarcinogenesis were confirmed by in vivo xenograft studies [99]. Hepatocellular carcinoma up-regulated long non-coding RNA (HULC) was first identified by a cDNA microarray as the most upregulated transcript in human HCC tissues [101]. HULC was shown to promote lipogenesis in HepG2 and Huh7 cells by inducing the methylation of CpG islands in the miR-9 promoter, resulting in the silencing of miR-9 [100]. As a result, there was an upregulation of miR-9 target peroxisome proliferator–activated receptor alpha (PPARA) and increase in PPARA target acyl-CoA synthetase subunit ACSL1 [100]. ACSL1-induced cholesterol production stimulated the proliferation of HCC cells. Interestingly, exogenous cholesterol upregulated HULC by a positive feedback loop, which involved the activation of the HULC promoter by retinoid x receptor (RXRA) [100]. lncRNA metastasis associated lung adenocarcinoma transcript 1 (MALAT1) functions as an oncogene in a variety of cancers, and it is overexpressed in HCC as well as in NASH patients [102,103]. MALAT1 was shown to interact with SREBP-1c to stabilize nuclear SREBP-1c protein facilitating steatosis [104].

### 4.3. Pathogenic Pathways and Processes

#### 4.3.1. Adipocyte Inflammation and Insulin Resistance

There is a direct correlation between the degree of insulin resistance, defined by a decreased responsiveness to insulin and characterized by reduced glucose disposal in non-hepatic tissues, and the severity of NASH [105]. Adipocyte dysfunctions in obesity, either genetic or induced by HFD, play an integral role in insulin resistance causing NASH [106]. White adipocytes synthesize, esterify and store TG in lipid droplets; they are extremely insulin-sensitive, and insulin functions by increasing glucose uptake, promoting the utilization of glucose for the production of glycerol and inhibiting lipolysis by lipases. Insulin acts by binding to the insulin receptor with the downstream activation of the PI3K/Akt pathway, and dysregulation of the mediators of the insulin signaling pathway is observed in adipocytes in obesity [107]. Adipokines, factors released from adipocytes, such as leptin and adiponectin, facilitate insulin sensitivity. Increased adipocyte inflammation is observed in obesity, which plays a key role in inducing insulin resistance [108]. Dietary glucose and fat induce inflammation by increasing oxidative stress and the activation of transcription factors, such as NF-κB, activator protein-1 (AP-1) and early growth response 1 (EGR1). Additional factors causing obesity-associated adipocyte inflammation include relative ischemia due to hypertrophy in adipocytes with the production of hypoxia inducible factor-1 (HIF-1), changes in the gut microbiome and an increase in gut permeability, facilitating the leakage of gut microbiome-produced inflammatory factors. Hypertrophic adipocytes release inflammatory factors, such as tumor necrosis factor α (TNFα), interleukin-6 (IL-6), resistin, C-C motif chemokine ligand 2 (CCL2/MCP-1) and plasminogen activator inhibitor-1 (PAI-1), resulting in the infiltration of inflammatory macrophages and immune cells, with the production of more inflammatory cytokines. These cytokines stimulate two major signaling pathways, JNK and NF-κB, which play a key role in inducing insulin resistance. JNK activation causes the serine phosphorylation of the insulin receptor substrate-1 (IRS-1), which inhibits the tyrosine phosphorylation of IRS-1 required for downstream insulin signaling, and JNK is also required for maintaining the pro-inflammatory function of macrophages [109,110]. NF-κB transcriptionally regulates a plethora of cytokines, adhesion molecules and transcription factors that contribute to insulin resistance [108]. These cytokines also inhibit adiponectin secretion, and indeed, reduced adiponectin and elevated TNFα and IL-6 are hallmarks of NASH. While insulin resistance in adipocytes increases lipolysis, there is simultaneous downregulation of transcription factors, such as PPARγ, ChREBP and FOXO1, which regulate the expression of genes necessary for TG biogenesis [107]. The net effect is an increase in circulating FFAs, which are taken up by the liver, esterified and stored as TG in lipid droplets, resulting in steatosis. The accumulation of lipids in the liver induces an inflammatory response, similar to that observed in adipocytes, with the activation of liver-resident macrophages, Kupffer cells, and the production of inflammatory cytokines, which further activates HSCs, inducing fibrosis and hence NASH.

#### 4.3.2. Gut Microbiome

A sedentary lifestyle along with changes in diet—especially the consumption of diet rich in saturated fats, sucrose and fructose—and the widespread use of antibiotics, both in humans and in farm animals, have resulted in a significant change in gut microbiomes, known as dysbiosis, contributing to the pathogenesis of many chronic diseases including NASH and cancer [111,112,113]. Additionally, the increased gut permeability associated with obesity leads to the leakage of bacterial metabolites and microbiota-associated molecular patterns (MAMPs) into the circulation to which liver is continuously exposed. In adult germ-free mice, the transplantation of feces from obese individuals resulted in increased body fat versus that from lean individuals, and co-housing with WT mice resulting in microbial transfer and caused an exacerbation of NASH in NASH-resistant mice [114,115]. In the gut microbiota of adult NASH patients, *Bacteroides* and *Ruminococcus* were more abundant and *Prevotella*, less abundant [116]. The increased abundance of alcohol-producing bacteria in the NASH microbiome was identified with a corresponding increase in blood ethanol levels, suggesting a potential role of alcohol-induced inflammation in these patients [117]. Comparison of microbiomes among healthy controls, NAFLD-related cirrhosis and NAFLD-related cirrhosis with HCC demonstrated that *Bacteroides* and *Ruminococcaceae* were increased whereas *Akkermanisa* and *Bifidobacterium* were reduced in NAFLD-HCC patients compared to in NAFLD only patients [118]. HCC patients also had higher circulating levels of inflammatory cytokines [118]. These studies demonstrate dysbiosis in NASH and NASH-HCC patients. Changes in metabolic pathways in the gut microbiota might contribute to the development of NASH-HCC, as described below.

Secondary bile acids are a major metabolite generated by dysbiosis, contributing to NASH and HCC. In the liver, primary bile acids, cholic acid and chenodeoxycholic acid, are synthesized from cholesterol by cytochrome P450 enzymes, and after conjugation with the amino acids glycine and taurine, they are secreted in the intestine following a meal. As a detergent, bile acids play an important role in lipid solubilization and digestion. Almost 90% of the conjugated primary bile acids are absorbed in the terminal ileum, and the remaining are dehydroxylated by the gut microbiota to unconjugated secondary bile acids, such as deoxycholic acid and lithocholic acid, which are reabsorbed to the liver via the portal vein. Mice fed a HFD accumulate Gram-positive bacteria that generate a secondary bile acid, DCA, as well as the MAMP lipoteichoic acid (LTA) [119,120]. The enterohepatic circulation of DCA and leakage of LTA due to increased gut permeability induces a senescence-associated secretory phenotype (SASP) in HSCs via Toll-like receptor 2 (TLR2), which secrete inflammatory and oncogenic factors to promote HCC [119,120]. Additionally, these HSCs also produce prostaglandin E2 (PGE2) via COX2 activation, which suppresses anti-tumor immunity and contributes to HCC progression [120]. Increased leakiness because of intestinal inflammation leads to high circulating levels of bacterial product lipopolysaccharide (LPS), inducing a chronic hepatic inflammatory response via the TLR-4 pathway, leading to HCC progression [121,122].

Bile acids play a major role in regulating metabolism by binding to Farnesoid X receptor (FXR) and controlling cholesterol levels, energy homeostasis, and glucose storage and release [123]. FXR inhibits *de novo* bile acid synthesis by inhibiting cytochrome P450 family 7 subfamily A member 1 (CYP7A1) and thus regulates lipid metabolism in hepatocytes, and it also regulates NF-κB activity. Fxr-/- mice develop NASH and HCC, and it was documented that the gut microbiota requires FXR to promote obesity and steatosis [124,125,126]. HFD feeding leads to the upregulation of Yin Yang 1 (YY1), which blocks FXR and thus promotes steatosis [127]. In non-cirrhotic NASH patients, a phase III trial with obeticholic acid, a semisynthetic primary bile acid, demonstrated an improvement in the histological features of NASH, suggesting its potential efficacy in NASH-HCC patients as well [128].

#### 4.3.3. Hepatic Inflammation and Immune Response

Intrahepatic chronic inflammation plays a central role in NASH and HCC, and the role of TNFα and IL-6, activating oncogenic STAT3 signaling, was demonstrated in dietary and genetic obesity-induced HCC development [129]. The transcription factor NF-κB plays a crucial role in inflammation by transcriptionally regulating a plethora of pro-inflammatory cytokines [130]. In the canonical pathway, the p50/p65 NF-κB heterodimer is sequestered in the cytoplasm by IκB. Pro-inflammatory signals activate IκB kinase (IKK), which phosphorylates IκB, resulting in its proteasome-mediated degradation and the translocation of p50/p65 to the nucleus, regulating gene transcription. IKK has two catalytic subunits, IKKα and IKKβ, bound to a regulatory subunit IKKγ, also known as NF-κB essential modifier (NEMO). The role of NF-κB in the liver is complex and cell-type specific. In the hepatocyte, it specifically functions as a survival factor and thus protects hepatocytes from DEN-induced injury, whereas it serves a pro-inflammatory function in macrophages. Indeed, knocking out IKKβ in hepatocytes promoted DEN-induced HCC while knocking it out in both hepatocytes and Kupffer cells protected against HCC development [131]. Knocking out NEMO in liver progenitor cells resulted in the spontaneous progressive development of NASH, cirrhosis and HCC, which could be prevented by antioxidants [132]. On the other hand, inflammasome activation in hepatocytes has been shown to play an important role in regulating NASH. The inflammasome is a multiprotein complex, comprised of NLR family pyrin domain containing 3 (NLRP3), PYD and CARD domain containing (PYCARD/ASC) and pro-caspase-1, which can be activated by saturated fatty acids or products of the gut microbiome, leading to the activation and release of caspase-1, which cleaves pro-IL-1β and pro-IL-18 to generate mature forms of the highly inflammatory cytokines IL-1β and IL-18 [133]. NLRP3, ASC and caspase-1 deficient mice were resistant to HFD-induced insulin resistance and NAFLD [134]. A small molecule inhibitor of NLRP3 could protect against NASH-associated inflammation and fibrosis in mice [135]. The role of the inflammasome in NASH-HCC remains to be studied.

NASH-induced HCC developed upon long-term feeding with a choline-deficient HFD via activated intrahepatic CD8+ T cells and natural killer T (NKT) cells that interacted with hepatocytes [136]. NKT cells induced steatosis primarily by secreting TNF superfamily member 14 (TNFSF14), also known as lymphotoxin β receptor ligand (LIGHT), which increases FA uptake in hepatocytes, [137] and NKT cells and CD8+ T cells cooperatively induced liver damage [136]. It was demonstrated that both TNFSF14 and canonical NF-κB signaling participate in the transition of NASH to HCC. However, studies using HFD fed MUP-uPA mice, in which hepatocyte ER stress was induced by plasminogen activator expression, and a STAM mouse model demonstrated the accumulation of immunoglobulin-producing (IgA+) plasmocytes that express programmed death ligand 1 (PD-L1) and IL-10 and suppress cytotoxic CD8+ T cells [138]. The ablation of CD8+ T cells accelerated HCC in these models, while the inhibition of IgA+ cells induced the regression of HCC in a CD8+ T cell-dependent manner [138]. On the other hand, there is selective loss of CD4+ T cells but not CD8+ T cells in NAFLD and NAFLD-mediated HCC [139]. The depletion of intrahepatic CD4+ T cells accelerated tumor development in Myc transgenic mice fed an MCDD. CD4+ T cells have higher levels of ROS, making them susceptible to FFA-generated ROS and selective loss, and antioxidants provided a protective effect [139]. These discrepant findings may be attributed to the types of models used and the underlying molecular mechanism and further stress the importance of using a model that faithfully mimics human obesity-induced NASH progressing to HCC. Although the MCDD model is commonly used and induces NASH, the mice lose body weight instead of gaining, so the findings from this model may not always provide the true picture [14]. Mice fed a high fat, high fructose diet develop NASH and, eventually, HCC and maintain a transcriptome profile similar to that of human NASH [140], and this model might be a good choice to perform in-depth immunological studies in the future. NK cell dysfunction in the tumor microenvironment causing the suppression of the anti-tumor immune response is associated with a higher rate of HCC development and progression and poor survival outcomes [141]. However, a recent study in NAFLD liver biopsies demonstrated the maintenance of NK cell number, phenotype and functionality [142]. These findings suggest that the dysfunction of NK cells might be a trigger for the switch from NASH to HCC, which needs to be experimentally validated.

#### 4.3.4. PI3K/Akt Signaling

Hyperinsulinemia and deregulated insulin signaling occur in obesity, type 2 diabetes and NAFLD, and insulin activates PI3K/Akt signaling, a key regulator of metabolism, cell growth and cell survival. PTEN is a negative regulator of the oncogenic PI3K/Akt pathway and acts by dephosphorylating phosphatidylinositol 3,4,5-triphosphate (PIP3), generated by PI3K. PTEN inactivation, by a variety of mechanisms including loss-of-function mutation and gene deletion, resulting in PI3K/Akt activation, is observed in ~40% HCC patients [143]. Hepatocyte-specific Pten knockout mice develop NASH, with an increase in SREBP-1c and lipogenic genes and eventually HCC [144]. PTEN levels were downregulated in NASH livers and in the livers of steatotic NASH, and it was documented that unsaturated fatty acids activate NF-κB/mTOR signaling, which induces miR-21, which directly targets PTEN [145,146]. It should be noted that liver-specific Akt2 knockout inhibited hepatic TG accumulation either in ob/ob mouse or upon feeding high fat diet (HFD), and a hepatocyte-specific Pik3ca transgenic mouse developed steatosis and HCC, further establishing the importance of the activation of the PI3K/Akt pathway in NASH and NASH-HCC [147,148].

#### 4.3.5. AMPK and Autophagy

AMP-activated protein kinase (AMPK) is activated by increased AMP levels and is an indicator of decreased cellular energy stores [149]. As such, AMPK activation stimulates energy generating catabolic pathways, such as FA β-oxidation, and inhibits anabolic processes such as fatty acid biosynthesis. The liver-specific activation of AMPK prevents hepatic steatosis, and AMPK activity is downregulated by inflammation, obesity and diabetes, suggesting that increasing AMPK activity might be a therapeutic strategy for NAFLD [150]. Indeed, the anti-diabetic drugs metformin and thiazolidinediones both activate AMPK [151]. AMPK is known to activate autophagy, a lysosome-dependent catabolic process in which cytoplasmic proteins and organelles are degraded to meet the specific energy demands of the cells, by phosphorylating and activating the autophagy initiating kinase ULK1 [152]. Autophagy plays a role in mobilizing lipids from lipid droplets, a process called lipophagy, and the inhibition of autophagy increased TG storage in the mouse liver and induced liver tumors [153,154]. In NAFLD, excess TG and FFAs suppress autophagy initiation by activating mTOR and inhibiting ULK1, resulting in increased oxidative stress. [152] As a counteractive measure, the transcription factor NRF2 (NFE2L2) is activated, leading to the activation of pro-survival genes, such as glutathione S-transferase and thioredoxin reductase 1 to neutralize the detrimental effects of ROS [155,156]. Activating mutations in the NFE2L2 gene are observed in human HCC patients, which stimulate cell proliferation [157,158].

## 5. Diagnostic Approaches Being Implemented

The diagnosis of obesity- and more precisely NASH-associated HCC is difficult [21]. NAFLD, in its early stages, is mostly asymptomatic [159]. Some patients complain of nonspecific symptoms such as fatigue while a few report pains in the right upper abdominal quadrant. Currently, no defined symptoms or physical examination findings can definitively diagnose NAFLD. Thoracic and abdominal imaging for non-liver symptoms revealing steatosis or the presence of abnormal liver function indicators in the blood along with absence of other etiologies, such as excessive alcohol intake, often point towards a diagnosis of NAFLD [160]. Additionally, there are a few clinical indicators for NAFLD, such as acanthosis nigricans arising from insulin resistance and a dorso-cervical hump, while with the onset of the compensated phase of cirrhosis, the patients might present with spider angiomata, palmar erythema, gynecomastia or prominent upper abdominal veins [159]. As cirrhosis becomes decompensated, with the loss of liver functions, more severe symptoms and signs develop, such as jaundice, ascites, splenomegaly, asterixis, portal hypertension and liver dysfunction, which calls for further investigation into the development of HCC. However, many NASH patients develop HCC without developing cirrhosis, further complicating the diagnosis process [161].

### 5.1. Liver Biopsy

For diagnosing NASH, a liver biopsy is the most definitive procedure because it provides information about the severity of steatosis, hepatocellular injury, inflammation and fibrosis, and can help in the determination of treatment options [159]. For HCC, a biopsy helps in diagnosis and does not contribute to decision making for the currently available treatment options [162]. However, a biopsy can definitely provide tumor tissues, which can be subjected to molecular analysis to identify biomarkers as well as dysregulations in molecular and signaling pathways to aid in precision medicine and treat the patients with appropriate targeted therapies. Numerous limitations limit the use of routine biopsies in NASH and HCC patients, which include invasive procedure-related complications, such as hemorrhage, patient anxiety and distress; sample- and observer-related variability; and most importantly, the potential of seeding tumor tissue [162,163]. Additionally, performing a liver biopsy on every patient suspected of having NASH is impractical. Therefore, a combination of methods, described in the next section, are used for the diagnosis of NASH.

### 5.2. Imaging

For the diagnosis of HCC, current guidelines recommend contrast-enhanced, cross-sectional imaging because of the sensitivity and specificity of the procedure to diagnose the tumor in the presence of cirrhosis [21]. For NASH, an abdominal ultrasound is a low-cost, low-risk, and widely available tool for diagnosis, although there are major limitations, such as the inability to distinguish NASH from NAFL and poor sensitivity to detect steatosis <30% [164]. Quantitative ultrasound technology (QUS) can now overcome those limitations and better characterize tissue microstructure by measuring fundamental acoustic parameters and can detect steatosis even in morbidly obese patients and identify steatosis [165]. However, the procedure suffers from operator-dependent subjectivity and qualitative interpretation variability.

Contrast computed tomography (CT) and magnetic resonance imaging (MRI) are more advanced imaging options. A Contrast CT scans have a sensitivity of 50–86% and a specificity of 75–87% to detect steatosis, while MRI can detect as low as 5% steatosis with almost 100% accuracy [166]. The employment of MRI definitely facilitates the early diagnosis of, intervention for and monitoring of the disease. However, both these procedures are expensive and not widely available, thus limiting the utilization of these approaches.

### 5.3. Liver Function Tests

Liver function tests are often abnormal in NASH patients. The levels of liver enzymes, such as aspartate aminotransferase (AST), alanine aminotransferase (AST) and/or gamma-glutamyltransferase (GGT), are often elevated, indicating liver injury, and markers of liver functions, such as albumin and prothrombin time, are altered with cirrhosis, along with an increase in serum bilirubin and decrease in platelet count [160]. However, these changes are insensitive in detecting early disease, and changes in these parameters are indicative of advanced disease complicated by the development of NASH.

### 5.4. Predictive Models

A number of low-cost, noninvasive predictive models, such as the NAFLD fibrosis score (NFS) and fibrosis score 4 (FIB-4), have been developed to assess fibrosis and identify patients who might benefit from a further biopsy [167]. For the NFS, metabolic risk factors such as age, body mass index and fasting glucose are evaluated along with clinical data such as the platelet count, albumin level, and ratio of AST to ALT. An NFS score above 0.676 indicates the presence of advanced fibrosis with 33% sensitivity and 98% specificity. For the FIB-4, age, AST, platelet counts and ALT are assessed as predictors of fibrosis.

### 5.5. Biomarkers of NASH and HCC

No single biomarker can accurately and consistently diagnose NASH. Cytokeratin-18 (CK-18) and terminal peptide of procollagen III (PIIINP) have been shown to associate with NASH [168]. A number of miRNAs that have been shown to play a role in NASH and HCC, such as miR-21, miR-221 and miR-122, can be detected in the peripheral circulation, especially in extracellular vesicles (EVs) such as exosomes [161]. However, their clinical application is yet to be standardized. The identification of circulating tumor DNA (ctDNA), circulating tumor cells (CTCs) and EVs in blood aids in not only the diagnosis of HCC but also patient stratification for treatment and monitoring treatment responses [21]. CTCs can be determined by the detection of the surface expression of epithelial cell adhesion molecule (EpCAM), cytokeratin, asiaglycoprotein receptor and carbamoyl phosphate synthase 1 (CPS1) in combination with the physical properties, DNA content and RNA signature [169,170,171]. CTC levels have been shown to correlate with tumor stage and outcome, and it was documented that mesenchymal CTCs were enriched in the peripheral veins of HCC patients, suggesting that the CTCs undergo EMT and acquire aggressive survival potential [172]. CTCs are also useful in detecting tumor heterogeneity, therapeutic sensitivity—e.g., pERK- or pAKT-positive CTCs can predict sorafenib sensitivity—and biomarkers for molecular stratification such as MET, FGFR4 and DNA-PK [169,173]. However, studies have shown that in HCC patients, very few CTCs exiting the liver via the hepatic vein and entering the pulmonary circulation actually reach the peripheral circulation, thus creating limitations in their detection [172]. A comparison with the serum metabolomic profiles of Mat1a knockout mice, which develop NASH-HCC, allowed the clustering of human NAFLD patients into two groups, one with serum metabolomic profiles similar to Mat1a knockout mice (M-subtype) and a second with different profiles (non-M-subtype) [174]. This analysis facilitated differentiation between steatosis and NASH in each subtype [174]. Plasma lipidomic analysis identified specific lipid species, such as sphingolipids and glycerophospholipids, to distinguish between steatosis and NASH [175]. As metabolomic analysis becomes more efficient and cost-effective, these comprehensive approaches might be used clinically for differential diagnosis of NASH and NASH-HCC in the future.

## 6. Therapeutic Intervention Strategies in Obesity-Associated HCC

### 6.1. Surgical Intervention

The management of HCC depends upon the stage of the disease as determined by the Barcelona Clinic for Liver Cancer (BCLC) algorithm, which incorporates liver function status; patient performance; and tumor number, size and spread [176]. Patients at early stage (BCLC-0 or BCLC-A) with preserved liver function and a single tumor < 5 cm or three tumors < 3 cm can be treated with liver transplantation or by the radiofrequency ablation (RFA) of the small tumors. Intermediate stage patients (BCLC-B) with preserved liver function and multifocal tumors without large vessel invasion can be treated with transarterial chemoembolization (TACE). However, both early and intermediate stage patients are very rare. The majority of NASH-HCC patients present with BCLC-C or BCLC-D stages with poor liver function and performance states and the presence of comorbidities, such as diabetes and cardiovascular diseases, and these patients are unsuitable for surgical intervention and are managed with medical treatment and supportive care.

### 6.2. Medical Management

For advanced stage HCC, the multi-kinase inhibitor sorafenib has been the treatment of choice for more than a decade, and recently, other tyrosine kinase inhibitors (TKIs) such as lenvatinib, regorafenib, cabozatinib and tivantinib have been approved, either as first line therapy or following sorafenib treatment [177,178,179,180,181]. Ramucirumab, a monoclonal antibody that blocks VEGF2R signaling, has also been approved for the treatment of advanced HCC [182]. However, none of these treatments provide a lasting impact on the overall survival of the patients, and it is also not clear whether there is a differential response between NASH-HCC patients versus HCC patients with other etiologies. Additionally, because of compromised liver function, drug-related toxicity is very high, with poor drug compliance in these patients. Insulin resistance is a hallmark of NASH, hence insulin sensitizers, such as PPARγ agonist thiazolidinediones (TZDs) and metformin, have shown efficacy in rodent models of NASH-HCC [183,184]. Metformin treatment showed a small but significant improvement in survival in patients with type 2 diabetes and HCC in a large study employing 5093 patients [185]. These strategies need further validation with a focus on NASH-HCC patients. As an antioxidant, vitamin E has been shown to protect against oxidative damage and improve inflammation and hepatocyte ballooning in NASH patients [186]. As yet, the efficacy of vitamin E in NASH-HCC patients remains to be determined. A meta-analysis revealed that use of cholesterol-lowering drug statins, which block HMG co-reductase, is associated with a reduced risk of HCC [187]. Statin use has a beneficial effect on NASH patients [188] and thus might benefit NASH-HCC patients as well, which needs to be evaluated in clinical trials.

A promising approach for HCC patients is immunotherapy, which includes immune checkpoint blockers/monoclonal antibodies against the programmed cell death protein 1 (PD-1), PD-1 ligand (PD-L1) and cytotoxic T lymphocyte antigen-4 (CTLA-4) such as nivolumab, pembrolizumab, MED14736, ipilimumab and tremelimumab [189]. The PD-1 inhibitor nivolumab was efficacious in ~20% of HCC patients of all etiologies with significantly improved survival benefits compared to TKIs, and nivolumab and pembrolizumab have been approved for HCC treatment as a second line therapy following sorafenib [190]. Initial studies with chimeric antigen receptor T cells targeting HCC antigen glypican 3 show promise in preclinical models, paving the way for the clinical use of this approach in the near future [191].

The current status of the different therapeutic agents being implemented are presented in Table 1.

### 6.3. Lifestyle Management

Since there are limited therapeutic options for NASH-HCC, active management should emphasize prevention and early detection. Preventive measures such as lifestyle modifications including diet and physical activity are very significant in this direction [194]. Relatively moderate weight loss (as low as 10% of body weight) has been shown to improve insulin resistance and reduce steatosis, while massive weight loss following bariatric surgery can ameliorate NASH with the reversal of cirrhosis [195]. Caloric restriction and a diet rich in monosaturated and polyunsaturated fatty acids, such as the Mediterranean diet, can thus have a major role in the management of not only NASH but also the early stages of NASH-HCC [196]. High iron absorption has been reported in NASH individuals, and hepatic iron deposition elevated the risk of HCC in NAFLD patients, suggesting taking precautions about iron intake [197,198]. Exercise not only improves cardiovascular health but also reduces steatosis, improves insulin sensitivity and modulates immune responses, promoting an anticancer immune microenvironment, and thus is an important component of lifestyle modification [199,200]. Regular exercise decreased HCC development in hepatocyte-specific PTEN knockout mice by increasing the phosphorylation of AMPK and decreasing the activity of mTOR [201].

## 7. Conclusions

There is accumulating epidemiological evidence linking obesity to HCC development, and obesity-associated HCC, impaired glucose tolerance and NAFLD are rising at an alarming rate. The main drawback in this context is the unavailability of adequate screening methods for early detection and the presence of an undefined population at an advanced stage who are at risk of developing HCC. As such, community education focused on lifestyle (increase in physical activity) and dietary (low carbohydrate/fat intake and high protein intake) modification has a huge role in preventing obesity, NASH and ultimately HCC.

**Figure 1:**
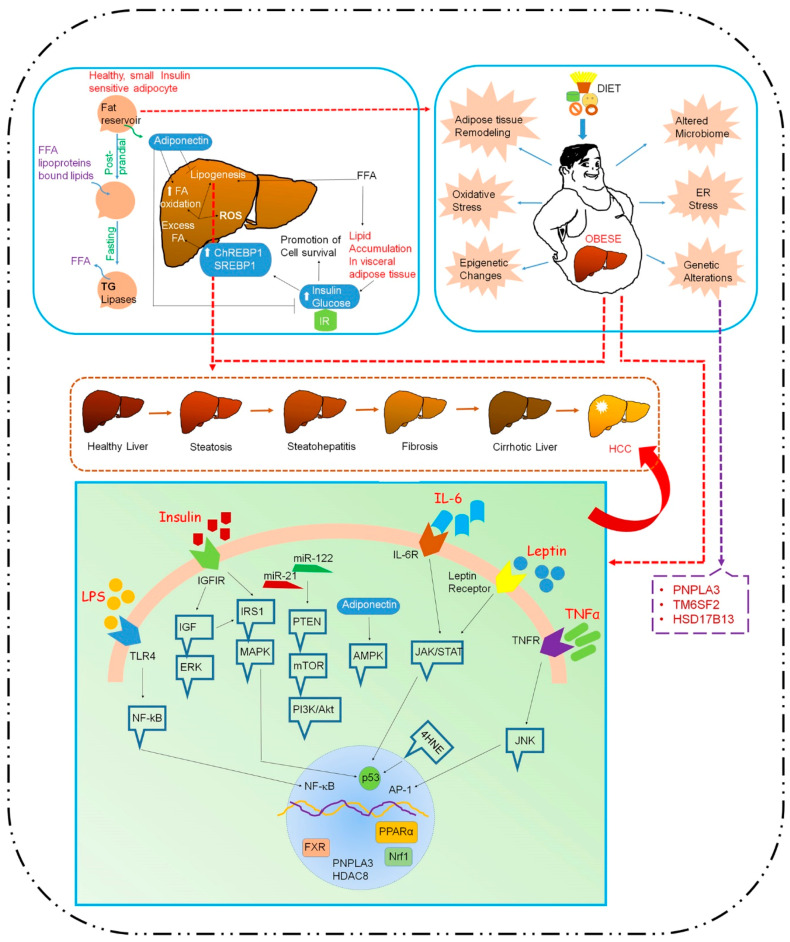
Molecular signaling pathways promoting HCC in the presence of obesity. Over-nutrition and a sedentary lifestyle induce adipose tissue remodeling, microbiome alteration, and ER and oxidative stress. These modifications, in association with genetic factors such as PNPLA3 and epigenetic changes, lead to the dysregulation of adipokine secretion and activation of the PI3K/Akt, JAK/STAT, NF-κB, mTOR, 4-HNE, and NRF-1 oncogenic pathways. Healthy adipocytes, in response to the above stimuli, absorb lipids and secrete adiponectin, which promotes insulin sensitivity and FA oxidation and suppresses lipogenesis. In the fasting state, adipocytes release FAs whereas in obesity, they swell and dedifferentiate, releasing less adiponectin. Subsequent macrophage infiltration contributes to inflammation. Lipolysis releases free fatty acids (FFAs), leading to triglyceride accumulation in VAT that generates IR. High FFAs and IR lead to steatosis, followed by hepatic lipogenesis by the transcriptional regulators SREBP1 and ChREBP1. Steatosis is mostly benign, but in the presence of excess FAs that are not converted into triglyceride, there is an overload of mitochondrial FA oxidation with the generation of ROS that promotes liver tissue damage and inflammation (NASH). IR facilitates high circulating glucose and insulin, which promotes cell survival and a tumor microenvironment. The persistent conditions promote DNA damage and HCC development. Additionally, obese adipose tissues promote an inflammatory response that contributes to liver damage, an impaired immune response and HCC progression. trans-4-hydroxy-2-nonenal (4-HNE); adenosine monophosphate activated protein kinase (AMPK); endoplasmic reticulum (ER); insulin-like growth factor-1 (IGF-1); interleukin-6 (IL-6); insulin receptor substrate-1 (IRS-1); mammalian target of rapamycin complex (mTOR); nuclear factor kappa B (NF-kB); nuclear factor erythroid 2 related factor 1 (Nrf-1); phosphatidylinositol-3 kinase (PI3K); PI3K/phosphatase and tensin homolog (PTEN); toll-like receptor (TLR); tumor necrosis factor alpha (TNFα); peroxisome proliferator activated receptor alpha (PPAR-α); fatty acid (FA); visceral adipose tissue (VAT); insulin resistance (IR); sterol regulatory element-binding protein (SREBP1); carbohydrate regulatory-binding protein (ChREBP1); reactive oxygen species (ROS); nonalcoholic steatohepatitis (NASH).

The external factors, specifically hyper-nutrition and a sedentary lifestyle, stimulate pathological changes in adipocytes and hepatocytes that significantly predispose to cancer progression (Figure 1). One of the key factors determining individual risk is genetic susceptibility, and a genomic survey can assist in identifying predisposing factors to recognize those at higher risk of developing disease. During disease progression, the analysis of circulating cytokines, miRNAs and tumor cells along with imaging techniques might aid in the early detection of HCC. An in depth unraveling of the mechanisms mediating disease progression, such as alterations in autophagy, the gut microbiome, bile acid synthesis and oxidative stress will facilitate the development of potential therapeutic strategies and also diagnostic biomarkers. Insulin-sensitizing drugs, such as statins, metformin and thiazolidinediones, should be considered as part of combinatorial pharmacotherapy and/or immunotherapy. A number of pharmaco-therapeutic agents have demonstrated some success, but research on more combinatorial therapeutic strategies and second-generation drug development will pave the way towards better treatment regimens for this challenging issue. NASH-HCC develops in a functionally compromised liver with a diminished capacity for drug detoxification, and as such, these patients are highly sensitive to drug toxicity. In this scenario, gene-based therapies provide a better alternative, especially because of high payload delivery to the target organ, the liver, following systemic administration. The identification of targets that regulate both NASH and HCC will therefore stimulate the development and evaluation of such therapies. Our recent studies have unraveled a novel role of AEG-1/MTDH, which is highly overexpressed in HCC patients and can function as a strong oncogene, in regulating both steatosis and inflammation, and a hepatocyte-specific nanoparticle delivering the AEG-1 siRNA showed pre-clinical efficacy in inhibiting HFD-induced NASH in mice and abrogated the growth of human HCC xenografts in nude mice, mandating further evaluation of these strategies in clinical trials [202,203].

In conclusion, identifying the right medication against obesity-associated HCC progression (target specific) for achieving long term treatment is appreciably under progress. Meanwhile, the most preventive and curative approach to be widely implemented is making efforts in lifestyle alterations (improving physical activity and modifying diet behavior), which, coupled with pharmacological assistance, will be a potential road to success.

## Figures and Tables

**Table 1:** Different pharmaco-therapeutic agents employed in obesity-associated hepatocellular carcinoma (HCC).

Pharmaceutical Agents	Target	Conditions	Current Status	Reference
Sorafenib and lenvatinib	Multiple tyrosine kinases	HCC of any etiology	Approved for first line therapy for advanced HCC	[177,178]
Regorafenib, cabozatinib and tivantinib	Multiple tyrosine kinases	HCC of any etiology	Approved for second line therapy following sorafenib for advanced HCC	[179,180,181]
Ramucirumab	Monoclonal antibody that blocks VEGF2R signaling	HCC of any etiology	Approved for second line therapy following sorafenib for advanced HCC	[182]
Nivolumab and pembrolizumab	PD-1 inhibitor	HCC of any etiology	Approved for second line therapy following sorafenib for advanced HCC	[190]
Statins	Endogenous cholesterol synthesis inhibitors targeting HMG-CoA reductase	NASH and related cardiovascular risk; Reduced risk of HCC	In clinical trials	[187,188]
Metformin	Activation of AMPK, inhibition of *de novo* lipogenesis and mTOR pathway	Insulin resistance, HCC	In clinical trials	[185,192,193]
